# Factors that influence psychiatric trainees’ choice of higher training specialty: mixed-methods study

**DOI:** 10.1192/bjb.2021.128

**Published:** 2023-06

**Authors:** Nicholas Wolstenholme, Iain McKinnon, Adrian J. Lloyd

**Affiliations:** 1 Tees, Esk and Wear Valleys NHS Foundation Trust, Darlington, UK; 2 Cumbria, Northumberland, Tyne and Wear NHS Foundation Trust, Morpeth, UK; 3 Newcastle University, Newcastle upon Tyne, UK; 4 Cumbria, Northumberland, Tyne and Wear NHS Foundation Trust, Newcastle upon Tyne, UK; 5 Health Education England, Newcastle upon Tyne, UK

**Keywords:** Education and training, qualitative research, specialty training, careers in psychiatry, workforce planning in psychiatry

## Abstract

**Aims and method:**

Factors influencing trainees’ decisions about choosing and remaining in higher training subspecialties have not been widely researched. We administered telephone questionnaires to higher specialist trainees in the north-east of England to ascertain what influences these decisions. Thematic analysis was employed to develop overall constructs.

**Results:**

Twenty-seven trainees were interviewed, resulting in six overall constructs. These were: supervisory experiences; perceived work–life balance; career prospects; training and working environments; interest in the chosen subspecialty; and previous experience within the chosen subspecialty. Most trainees interviewed felt they had made the right specialty choice.

**Clinical implications:**

This study demonstrates the particular importance of exposure to a specialty and perceptions of the supervisory experience in determining trainees’ choices of, and decisions to remain in, a particular psychiatric specialty. Factors highlighted in this study must inform training, recruitment and workforce planning in order to bolster the recruitment and retention of trainees into higher specialty training.

UK medical doctors pursuing psychiatry careers usually apply to core psychiatry training following completion of foundation programmes (the first 2 years of postgraduate medical practice). Core training comprises six 6-month placements in various psychiatric specialties. This leads to the Royal College of Psychiatrists’ Membership examinations (MRCPsych), allowing trainees to apply competitively to one of six psychiatric specialties defined by the UK General Medical Council and the RCPsych's curriculum^1^ or to certain combinations of these. The three stages (years) of higher specialist training are denoted ST4–ST6, the first three stages having been completed in core training (years CT1–CT3). After completing 3 years (more if dual training), a Certificate of Completion of Training is awarded, allowing entry to the UK specialist register.

Psychiatry has struggled with recruitment into both core and higher training, although core training had near-full recruitment across the UK in 2020.^2,3^ However, under-recruitment to psychiatry higher specialist training continues.^4^ The six UK psychiatric specialties (referred to in this paper for clarity as subspecialties) are: general adult; old age; child and adolescent; forensic; medical psychotherapy; and psychiatry of intellectual disability.^1^ Additionally, some dual training places exist, combining two specialties into a joint higher training scheme.^5^ Those with the consistently best recruitment between 2017 and 2020 (data for England, Scotland and Wales combined) were forensic (66–78% fill) and dual general adult/psychotherapy (67–92%). Child and adolescent, general adult, old age and dual general adult/old age held a mid-ground of 48–69% fill; psychiatry of intellectual disability had the lowest fill rate, at 29–36%. Medical psychotherapy alone and other dual training combinations comprise low numbers, making percentage fill rates difficult to interpret. The 2020–2021 recruitment year (coincident with the COVID-19 pandemic) had notably increased recruitment to most of the schemes mentioned above, although general adult and intellectual disability fill rates were static (69% and 33% respectively).

Recruitment and retention has been researched broadly,^6–8^ often with a focus on the initial choice of psychiatry at core training level^9^ and to a very limited extent within individual higher psychiatric specialties.^10,11^ Little information is available regarding the factors influencing psychiatry trainees’ choice between subspecialties at the midpoint of their psychiatric training.

## Method

A mainly qualitative methodology, employing semi-structured interviews, was used to investigate attitudes and decision-making.

Study approval as an educational project was obtained from the lead employer for trainees in the north-east of England (Northumbria Healthcare NHS Foundation Trust) and two NHS ʻhost' trusts: Tees, Esk and Wear Valleys (TEWV) and Northumberland, Tyne and Wear (NTW) NHS Foundation Trusts (the latter has since become Cumbria, Northumberland, Tyne & Wear NHS Foundation Trust). Health Research Authority/ethical approval was not required.

A list of higher trainees in the Health Education England North East and North Cumbria (HEENE) region was obtained. Owing to the wide geographical spread, telephone interviews were approved by the relevant organisations. Higher trainees within the first 6-months of training were excluded as they had limited experience of their chosen subspecialty.

Questions explored demographics, decisions to apply to the subspecialty and views on continuing in it (supplementary Appendix 1, available at https://doi.org/10.1192/bjb.2021.128). A participant information sheet and consent form were provided by email. Reminder emails were sent every 2 weeks for 2 months if there was no response. Following written or verbal consent, telephone interviews were conducted by N.W., each lasting 15–30 min, between June and September 2017. Final-year higher trainees were prioritised as they were approaching the completion of training. Responses were transcribed in real time by N.W., containing only demographic information and anonymised numerical identifiers. Identifiable data were permanently deleted prior to data archiving.

Transcripts were analysed using thematic analysis.^12^ Data-sets were coded in Microsoft Word and sorted manually. Co-coding of a subset of transcripts took place with a clinical educationalist (A.L.), which helped stimulate discussion and allowed improved contextualisation and reliability of the data. During analysis, codes were continually reviewed and collated into emergent themes; groups of higher-level themes were identified using the constant comparative method.^13^ Original codes were reviewed again with reference to the original data-sets. Examples that illustrated higher themes were extracted from trainee responses. Overall constructs were then developed from higher themes.

## Results

### Description of the sample

Twenty-seven (71%) of thirty-eight eligible trainees identified took part in the study. One did not wish to take part and ten did not respond. Table 1 shows the demographic data of respondents. Most (85%) had at least a year's experience of higher training, with (96%) training in their first-choice subspecialty.

**Table 1:** Demographic data and description of the sample

Gender, *n* (%)	
Male	7 (26)
Female	20 (74)
Age, years	
mean (s.d.)	37.7 (6.2)
median (Q1, Q3, IQR)	36 (33, 41.5, 8.5)
Country/region of graduation, *n* (%)	
UK	15 (56%)
EU	2 (7%)
Non-EU	10 (37%)
Medicine was first degree, *n* (%)	
Yes	24 (89%)
No	3 (11%)
Straight into psychiatry from foundation training, *n* (%)	
Yes	16 (59%)
No	11 (41%)
Psychiatry was first-choice specialty, *n* (%)	
Yes	19 (70%)
No	8 (30%)
Subspecialty, *n* (%)^a^	
General adult	9 (33%)
Old age	6 (22%)
Child and adolescent	6 (22%)
Intellectual disability	2 (7%)
Psychotherapy	1 (4%)
Dual training	3 (11%)
Forensic	0 (0%)
Level of training, *n* (%)^b^	
ST4	4 (15%)
ST5	12 (44%)
ST6	11 (41%)
Psychiatric subspecialty was first choice, *n* (%)	
Yes	26 (96%)
No	1 (4%)

Q, quartile; IQR, interquartile range.

a. Cumulative percentage is 99%: this is an artefact of rounding effects.

b. ST4–ST6 (Specialty Trainee Years 4–6) are the 3 years of psychiatric higher training. Earlier years are ‘core training’ (CT1–CT3).

### Results of initial screening questions

Most trainees (71%) felt certain or partially certain that they had sufficient exposure to all subspecialties when making their choice. A good core training experience played a significant (70%) or partial (19%) role in their decision. Approximately half of trainees felt that lifestyle factors were significant, with most trainees (93%) happy within their choice of subspecialty; 78% said it had lived up to their expectations (supplementary Appendix 2).

### Results from the thematic analysis

**Fig. 1:**
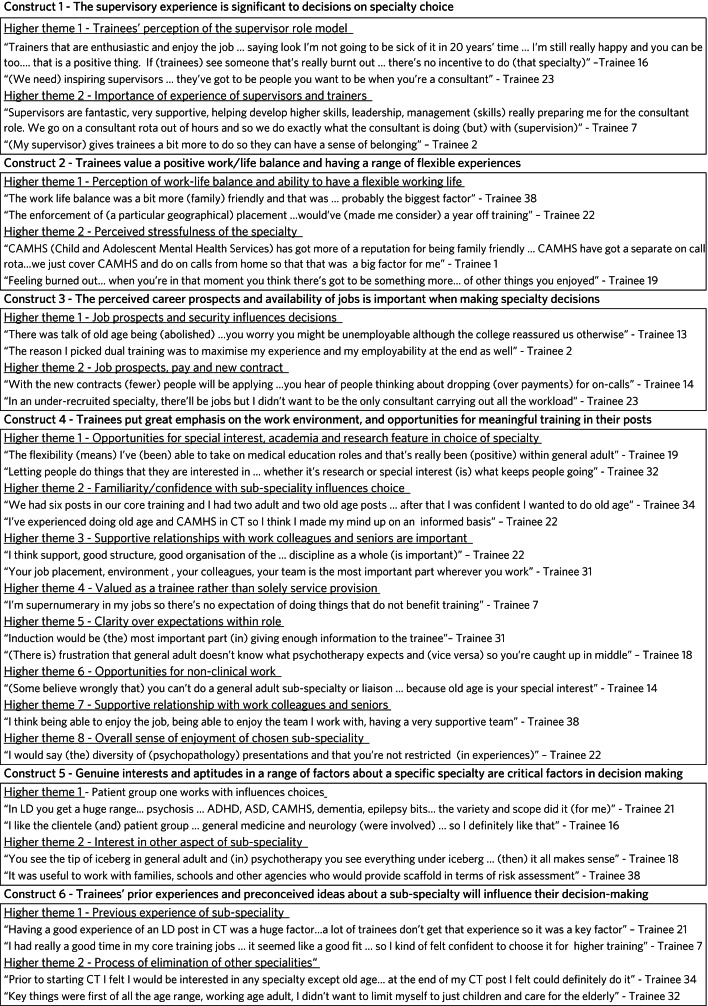
Overall constructs, supporting higher themes and supporting data. CT, core trainee; CAMHS, child and adolescent mental health services; CMHT, community mental health team.

The 27 interviews yielded 388 codes, sorted into 48 initial themes. Twenty higher-level themes were developed, with six overall constructs eventually derived from the data analysis (supplementary Appendix 3). Four constructs were common to trainees’ decisions both to apply to and continue in higher training specialties: ʻsupervisory experience', ʻperceived work–life balance', ʻcareer prospects' and ʻtraining/working environment'. With respect to applying for a subspecialty, additional constructs of ʻinterest in subspecialty' and ʻprevious experience in subspecialty' were characterised. Construct development and source material are presented in Fig. 1.

#### Supervisory experience (Fig. 1, construct 1)

Good supervisory experience was consistently stated to be an important indicator for making future decisions about subspecialty choice and staying within a specialty. Opinions were reported about both perceived positive and negative core training placements. Supervisors appear to influence trainees in various ways; some felt that the supervisory environment gave a sense of belonging, encouragement and support:

ʻI had an excellent supervisor on my ST4 post who was really quite supportive, encouraging and [ … ] gave me more confidence. [With] a less good supervisor that might not have been the case' (Trainee 24).

Others cited the importance of how supervisors approach trainees’ maturational development:

ʻ … trainers I've had have been excellent, really enthusiastic [ … ] treating me like an adult and also training you to be a consultant [ … ] more like an apprenticeship [ … ] you feel like you are on a road to somewhere' (Trainee 21).

The supervisor's attitude towards their own subspecialty also influenced trainees:

ʻI had a fabulous supervisor [ … ] I think that he was infectious and very enthusiastic in training' (Trainee 16).

Some held their supervisors as role models influencing where they see themselves working in the future, whereas poor supervisory experiences appeared to have a detrimental impact on choices:

ʻI know of CTs [core trainees] who have left training programmes not directly due to the supervisor but the supervisor hadn't helped' (Trainee 24).

#### Work–life balance and range of experiences (Fig. 1, construct 2)

Trainees expressed views on the perceived work–life balance of their specialty. Some had preconceptions about particular subspecialties being more ʻfamily-friendly' or ʻslower paced' than others, and several trainees noted work–life difficulties in their current subspecialty, which may have influenced them negatively:

ʻI often feel I had to sacrifice way too much for what I get out of it. There are so many demands and so little time for me' (Trainee 18)

‘They've brought in night shifts for higher trainees … that's something I very much dislike (Trainee 13).

#### Career prospects (Fig. 1, construct 3)

Trainees were concerned about the availability of substantive consultant posts at the end of training:

ʻIn terms of professional development and employment over a long career you need to be able to see reliable jobs at end of the training process' (Trainee 34).

Old age psychiatry trainees, or those considering it as a choice, expressed concerns about the introduction of ʻageless services' that was being discussed around the time of the survey, which they feared might make their specialty obsolete. Conversely, trainees from other subspecialties voiced concerns about job availability, under-recruitment and the potential impact on workload:

ʻLD [learning/intellectual disability] is pretty specialised. [The UK is] the only country in the world that has it as a set specialty and because it is undersubscribed they don't get a lot of trainees' (Trainee 21).

For some trainees, employment opportunities at the end of training played a key role in their decision to choose dual training.

#### Training/working environment (Fig. 1, construct 4)

The working environment, relationships with colleagues and their team were important factors in choosing and continuing in a subspecialty. Where trainees felt to some degree supernumerary in their roles, this was perceived to be important in allowing them to develop skills outside main clinical competencies:

ʻ[Look] after your trainees and [make] sure they have a good training experience and they feel like they're part of a training post rather than just [providing service]' (Trainee 27).

General adult psychiatry trainees appreciated the flexibility of having special interest and medical education opportunities:

ʻI knew that [ … ] I'd have options of more clinical special interest sessions [ … ] branching into other special interest sessions like perinatal' (Trainee 37).

Some trainees from other subspecialties perceived that these options were somehow not available to them.

#### Interest in subspecialty (Fig. 1, construct 5)

Some trainees reflected on the aspects within their subspecialty that led them to apply, including being able to see patients getting better, the opportunity to build skills and the range of experiences specific to that trainee's area of interest:

ʻI worked in the crisis team and you can see how patients respond really fast and that was quite rewarding' (Trainee 11)

ʻI think probably the holistic approach to the patients and I think in my case I liked the medicine because of my background, I find this specialty more appealing' (Trainee 31).

Some felt their interest was more specifically related to the particular patient group or the families involved:

ʻWorking with vulnerable people with great difficulties in various aspects of life, cognition, cognitive impairments and different mental disorders' (Trainee 31).

One trainee expressed the importance of variety within the workload and some trainees had had specialty interests since their undergraduate studies.

#### Previous experience in subspecialty (Fig. 1, construct 6)

Many trainees had a good training experience prior to their higher training, again linked to supervisory experience:

ʻI had an excellent core training experience that [ … ] made me want to do old age more' (Trainee 23).

Several trainees stated that a positive core training experience had resulted in them choosing their subspecialty when, prior to this, they had assumed they would dislike it:

ʻI hadn't wanted to do CAMHS [child and adolescent mental health services] as a rotation in core training but I was forced to do a rotation in CAMHS and I loved it' (Trainee 20).

Trainee 18 described a ʻlightbulb moment' during foundation training which appears to have led to a long-term interest and choice of subspecialty. Another trainee contrasted their positive training experience with observed negative experiences for other trainees:

All they do is get thrown on ward, go and get history from a chap who has already given twenty histories … if that was my experience of psychiatry I would never have wanted to do it either' (Trainee 2).

## Discussion

Most psychiatric trainees interviewed were happy with their choice of subspecialty and in the main, it had lived up to their expectations. Trainees attached substantial importance to some common themes for choosing, and remaining in, their subspecialty. Supervisory experience was significant at all levels of training, an observation consistent with those of other authors.^8,14^ Supervisors were viewed as role models and trainees used supervisory examples to gauge whether they could see themselves in that role, suggesting a need for high-quality supervision throughout training. A good core training experience was another influential factor and of particular importance in recruitment, as many trainees report using a process of elimination to choose a higher training subspecialty. This reflects conclusions regarding factors influencing the choice of psychiatry at earlier career points.^9^ Furthermore, a genuine interest in the subspecialty, its patients and the prospect of making a difference to them was present in the data.

Views on lifestyle factors appeared polarised, with around half of trainees stating it had no influence on their decision-making. For others it was significant, or even the most significant factor in subspecialty choice. Responses suggest that some trainees may have preconceptions, voiced by colleagues, that particular subspecialties are ʻquieter', ʻmore family-friendly', with ʻless risk' or ʻlighter on-call commitments'. It is therefore important that core training offers a wide range of high-quality training posts so trainees can make decisions based on first-hand experience.

General concerns existed around consultant employment prospects, and that hard-to-fill subspecialties had greater workloads because of gaps in service. Some trainees chose to dual-train to maximise career opportunities. Discussions about ʻageless services’^15,16^ may have affected trainees’ attitudes towards old age psychiatry as a career, leading to consideration of dual training or other subspecialties, with a negative knock-on effect on recruitment.

A study of UK psychiatric trainees’ progression found that only around 15% completed core and specialty training within the minimum 6 years, despite two-thirds completing core training within 3 years.^14^ The greatest discontinuity to progression was between the final year of core training (CT3) and the first year of higher specialist training (ST4). The vast majority of trainees did eventually complete training, entering the UK Specialist Register, but by a less direct route. Thus, choices made at that transition point appear to be crucially important in recruitment and retention in subspecialties.

There is limited literature about career choices at this transition point, with no published research found exploring subspecialty choices after CT3. As with our data, Parvez & Stewart noted the positive influence of role models and supportive colleagues on trainees’ decisions to apply and continue in their subspecialty.^11^ They, and McAlpine and colleagues,^17^ also found similar themes relating to the perceived future of old age psychiatry: service cuts and job insecurity. A recent survey of intellectual disability trainees highlighted personal or working life factors as important in choosing that specialty, but professional isolation was a concern.^18^ As we had no forensic psychiatry respondents, is it not possible to compare findings directly with those of Lowe & Hynes,^10^ who investigated core trainees’ views on choosing forensic higher training.

Parallel themes have emerged in studies exploring medical students’ choice of psychiatry as a career. Positive role models and early exposure to psychiatry are highlighted in a qualitative study of trainees in London, UK.^19^ A study in Swansea, UK, found that early exposure improved perceptions of psychiatry among undergraduates but did not change stated career intentions,^20^ and a Canadian study found that duration of psychiatry placement was a predictor of psychiatry becoming the preferred discipline.^21^ There has also been substantial work carried out by the RCPsych to improve the guidance given to medical schools, with the intention of improving the standing of psychiatry as a career choice for undergraduates.^22^

### Limitations of the study

Transcripts were typed in real time while conducting interviews via a headset. N.W. is able to touch type at the necessary speed, but this does raise the possibility that subtle aspects of interviews were missed. Telephone interviews were approved for this study, although video calls may have yielded further subtleties in non-verbal communication. Coding and thematic analysis were performed manually, although this could have been enhanced by access to digital analysis software.

Data were collected prior to North Cumbria psychiatry posts being transferred from Health Education England (HEE) North-West to HEE North East, hence the following descriptions are given for the HEE-NE area only for consistency. The North East region of England is geographically large (8579 km^2^; population just over 2.6 million), with a mix of urban centres and large rural areas.^23^ Psychiatry training is provided mostly within two large specialist mental health NHS trusts, to almost 100 core and 73 higher trainees. Recruitment during participants’ years of appointment largely followed national trends, although North East fill rates were lower than the national average in general adult, old age and intellectual disability.^4^ Although one region and a relatively small sample, this study builds on work done in other parts of the UK and, importantly, explores higher specialty choices at an under-researched stage of career progression. Thus, it begins to inform understanding and possible future research areas. It also informs employers of how trainees’ perceptions of current consultants’ workloads or feelings towards their jobs can significantly affect career choices.

We considered including final-year core trainees in the study, but interview questions would have differed substantially, therefore lacking comparability with the higher trainee data. We also excluded trainees in the first 6 months of higher training, as we felt they would not be able to adequately comment on questions about continuing on their training path. The age distribution of participants clustered in the late 30s, which appeared initially to be older than expected. Taking into account that trainees progressing directly from medical school to higher training would be 28 years old, combined with data from Medisauskaite et al^14^ suggesting slower progression for many trainees, this may not be an atypical picture. However, interrogation of national age data would be required to make any definitive judgements.

Focus groups were considered as an alternative to individual interviews; these may have generated more subthemes but this project did not have funding to support them. Furthermore, we judged that individual interviews might permit trainees freedom to give franker accounts. Aggregated data showed that there were responses from all subspecialties except for forensic psychiatry, although the subspecialty of individual interviewees was not analysed in order to preserve anonymity.

During initial theme development, some themes were chosen despite being represented by relatively low numbers of codes because of their importance to the topic matter. However, as analysis progressed, saturation was felt to be reached when no new codes were found in the data.

### Future research

To our knowledge, this is the only paper exploring a range of higher subspecialties within psychiatry. Future studies should employ more purposive sampling to ensure inclusion of all subspecialties.

## Data Availability

Anonymised data can be made available by the corresponding author on reasonable request.

## About the authors

**Nicholas Wolstenholme** is an ST6 psychiatric trainee with Tees, Esk and Wear Valleys NHS Foundation Trust, Darlington, UK. **Iain McKinnon** is a consultant psychiatrist with Cumbria, Northumberland, Tyne and Wear NHS Foundation Trust, Morpeth; and an honorary senior clinical lecturer in the Population Health Sciences Institute at Newcastle University, Newcastle upon Tyne, UK. **Adrian Lloyd** is a consultant psychiatrist with Cumbria, Northumberland, Tyne and Wear NHS Foundation Trust, Newcastle upon Tyne; and an honorary senior clinical lecturer in the Translational and Clinical Research Institute at Newcastle University, Newcastle upon Tyne; and Head of the School of Psychiatry, Health Education England: North East and North Cumbria, Newcastle upon Tyne, UK.

## Supplementary material

For supplementary material accompanying this paper visit https://doi.org/10.1192/bjb.2021.128. click here to view supplementary material
