# Supplementary material for: Factors that influence psychiatric trainees’ choice of higher training specialty: mixed-methods study
Source: BJPsych Bull. 2023 Jun;47(3):171–7. doi: 10.1192/bjb.2021.128 (PMC10214430; doi:10.1192/bjb.2021.128)
Supplement: Supplementary file 1 [file bjbsup.zip › S2056469421001285sup001.docx]

# Appendix 1 – Trainee Questionnaire

| Initial screening questions | |
| --- | --- |
| - What is your gender? - What is your age? - What is your higher training sub-specialty? Is it dual training? - What level of training are you currently at? - Are you a UK/EU/non EU graduate? - Was medicine your first degree? - Did you go into Psychiatry straight after foundation training? - Was Psychiatry your first choice specialty? - Was your Psychiatric sub-specialty your first choice? |  |

| Interview Questions - Applying to chosen sub-specialty |
| --- |
| - Before you applied to higher training did you feel you’d had sufficient exposure to all the sub-specialties in order to help you make your decision? Yes/No/Partially/Uncertain.   - FOLLOW UP PROMPT: Tell me more about that. - Have a think back to when you started thinking about your Psychiatry sub-specialty as a career. What do you think were the key things that attracted you to this sub-specialty?   - PROMPT: Were there any other factors that steered you towards it? - What would you say was the single most important factor was?   - PROMPT: Why was that? - To what degree did a good Core Training placement experience play a part in your decision to choose your sub-specialty? Significant/It didn’t play a part/partial.   - FOLLOW UP PROMPT: What where the particular aspects that were important in Core Training? - To what degree did lifestyle factors play a part in your decision to choose your sub-specialty? Significant/Partial/Small (or didn’t)   - FOLLOW UP PROMPT: Were there any particular examples that were important to you? - Was there anything about the sub-specialty that nearly put you off considering it, at that early stage?   - PROMPT: Why was this? - Were there any obstacles in your way in obtaining training in this sub-specialty?   - PROMPT: Why do you see these issues as obstacles? Any facilitators? - Overall, what factors do you think are most important for encouraging recruitment to your sub-specialty?   - PROMPT: How would these factors work to improve recruitment? |
| Interview Questions – Continuing in chosen sub-specialty |
| - Are you happy with your choice of sub-specialty? Yes/No/Uncertain   - PROMPT: Are there areas that you are more / less satisfied with? - Has it lived up to your expectations? Yes/No/Partially   - PROMPT: What particular aspects have met / not met what you were expecting? - Has there been anything since starting your sub-specialty training which has made you think about discontinuing this (e.g. switching to another sub-specialty)?   - PROMPT: What has prevented you from doing so / what has facilitated a decision to do so? - Once you had started training in your sub-specialty, what do you think have been the most important factors in encouraging you to continue?   - PROMPT: How have these factors impacted on your decision making? - Overall, what factors do you think are most important for ensuring that people continue in your sub-specialty?   - PROMPT: How could these factors be introduced into / maintained within training? - Thanks very much indeed for completing the survey. Do you have anything further to add that you think might be useful? |
