# Supplementary material for: Factors that influence psychiatric trainees’ choice of higher training specialty: mixed-methods study
Source: BJPsych Bull. 2023 Jun;47(3):171–7. doi: 10.1192/bjb.2021.128 (PMC10214430; doi:10.1192/bjb.2021.128)
Supplement: Supplementary file 1 [file bjbsup.zip › S2056469421001285sup002.docx]

| Appendix 2 – Results of initial screening questions |
| --- |
| **Before you applied to higher training, did you feel you had sufficient exposure to all the sub-specialties in order to help you make your decision?**   - Yes 14 (52%) - No 6 (22%) - Partially 5 (19%) - Uncertain 2 (7%)   **To what degree did a good Core Training placement experience play a part in your decision to choose your sub-specialty?**   - Significant 19 (70%) - It didn’t play a part 3 (11%) - Partial 5 (19%)   **To what degree did lifestyle factors play a part in your decision to choose your sub-specialty?**   - Significant 8 (30%) - Partially 6 (22%) - Small (or didn’t) 13 (48%)   **Are you happy with your choice of sub-specialty?**   - Yes 25 (93%) - No 0 (0%) - Uncertain 2 (7%)   **Has it lived up to your expectations?**   - Yes 21 (78%) - No 0 (0%) - Partially 6 (22%) |
