# Supplementary material for: Factors that influence psychiatric trainees’ choice of higher training specialty: mixed-methods study
Source: BJPsych Bull. 2023 Jun;47(3):171–7. doi: 10.1192/bjb.2021.128 (PMC10214430; doi:10.1192/bjb.2021.128)
Supplement: Supplementary file 1 [file bjbsup.zip › S2056469421001285sup003.docx]

# Appendix 3. Stages of the thematic analysis

| **Stage** | **Transaction** | **Outcome** | |
| --- | --- | --- | --- |
|  |  | **Applying to subspecialty** | **Continuing in subspecialty** |
| **1**  **↓** | Two datasets collected between June and September 2017 | 27 questionnaires | 27 questionnaires |
| **2** | Coding of verbatim responses | 243 codes obtained | 145 codes obtained |
| **↓** |  |  |  |
| **3**  **↓** | Sorting of codes into initial themes | 33 initial themes identified | 15 initial themes identified |
| **4**  **↓** | Higher level themes developed from initial themes | 11 higher level themes identified | 9 higher level themes identified |
| **5** | Review of codes against transcripts and assignment of themes into constructs | Six overall constructs | Four overall constructs |
